# Natural polysaccharides as modulators of mitochondrial homeostasis: structural determinants, pharmacological mechanisms, and translational challenges

**DOI:** 10.3389/fphar.2026.1948381

**Published:** 2026-10-14

**Authors:** Xinfang Du, Jingwen Zhang, Wenjing Li, Qinying Li

**Affiliations:** 1 Clinical Research Center, Affiliated Hospital of Shandong Second Medical University, Weifang, China; 2 Department of Endocrinology and Metabolism, Affiliated Hospital of Shandong Second Medical University, Weifang, China

**Keywords:** mitochondrial dysfunction, mitophagy, natural polysaccharides, oxidative stress, structure-activity relationship, therapeutic applications

## Abstract

Mitochondrial dysfunction is a convergent pathological feature of neurodegenerative, metabolic, cardiovascular, hepatic, and cancer-related disorders, making mitochondrial homeostasis an important therapeutic target. Natural polysaccharides (NPs) are structurally diverse macromolecules that have emerged as promising multifunctional modulators of mitochondrial homeostasis. This review summarizes the structural basis and mechanistic actions underlying the mitochondrial regulatory effects of NPs. Key structural features, including molecular weight, monosaccharide composition, glycosidic linkages, branching patterns, and chemical modifications, largely determine the mitochondria-regulating potential of NPs. Mechanistically, NPs regulate mitochondrial homeostasis by restoring bioenergetic function, reducing oxidative stress, maintaining mitochondrial quality control, and modulating mitochondria-dependent apoptosis. These effects support disease-context-specific therapeutic benefits, as NPs generally preserve mitochondrial homeostasis in non-malignant diseases while potentially inducing mitochondrial stress and cell death in cancer. This review also highlights key translational barriers to NP-based mitochondrial therapy, underscoring the need for standardized structural characterization, mechanism-oriented mitochondrial assays, and disease-specific evaluation.

## 1 Introduction

Mitochondria are indispensable organelles for cellular homeostasis. They serve not only as the primary sites of adenosine triphosphate (ATP) production but also as key regulators of redox signaling, calcium buffering, intermediary metabolism, and cell death (Suomalainen and Nunnari, 2024). Beyond their bioenergetic function, mitochondria act as dynamic signaling hubs that sense metabolic and environmental stress and coordinate adaptive responses at both cellular and tissue levels. Accordingly, mitochondrial integrity is essential for physiological function, particularly in highly energy-demanding and stress-sensitive organs.

Mitochondrial dysfunction has been increasingly recognized as a convergent pathological feature of human diseases. It is commonly characterized by impaired oxidative phosphorylation (OXPHOS), excessive reactive oxygen species (ROS) accumulation, disrupted mitochondrial dynamics, defective mitophagy, and calcium dyshomeostasis (Harrington et al., 2023). These abnormalities are highly interconnected and can form self-amplifying pathological loops that promote metabolic imbalance, inflammatory activation, and cell death. Such alterations contribute to the onset and progression of neurodegenerative, metabolic, cardiovascular, hepatic, and malignant diseases (Xu et al., 2025). Therefore, strategies aimed at restoring mitochondrial homeostasis have attracted increasing therapeutic interest.

Despite substantial progress, currently available mitochondrial interventions remain limited. Most existing approaches rely on synthetic small molecules or single-target modulators, which often show restricted selectivity, unsatisfactory biocompatibility, and limited efficacy in complex mitochondrial injury (Zong et al., 2024). In complex diseases, mitochondrial dysfunction is often driven by interconnected pathological processes, underscoring the need for therapies that modulate multiple aspects of mitochondrial homeostasis.

Natural polysaccharides (NPs), a diverse class of macromolecules derived from plants, fungi, algae, and animals, have attracted increasing attention for their potential in mitochondria-related therapy (Zhang Z. et al., 2022). Their structural diversity, favorable biocompatibility, and low toxicity provide a strong basis for therapeutic development. Functionally, NPs can improve respiratory-chain activity and energy metabolism, attenuate oxidative stress, regulate mitochondrial dynamics and mitophagy, and preserve mitochondrial membrane stability and calcium homeostasis (Chen et al., 2023; Rahmani et al., 2024). These broad regulatory effects suggest their potential for treating diseases driven by complex mitochondrial dysfunction.

In this review, we present a structure–mechanism–application perspective on the mitochondrial regulatory effects of NPs. Unlike previous reviews mainly focusing on the general antioxidant or anti-inflammatory activities, this review emphasizes the links among NP structural features, mitochondrial homeostasis, and disease-context-specific therapeutic implications. We also discuss current challenges and future directions for developing NPs as mitochondria-regulating therapeutic candidates.

## 2 Methods—literature search strategy

A targeted literature search was conducted in PubMed, Web of Science, Scopus, ScienceDirect, and Google Scholar up to July 2026. Search terms included combinations of “natural polysaccharides”, “mitochondria”, “mitochondrial dysfunction”, “oxidative phosphorylation”, “oxidative stress”, “mitophagy”, “mitochondrial dynamics”, “calcium homeostasis”, and “apoptosis”, together with terms related to the disease categories covered in this review. Original *in vitro*, *in vivo*, and clinical studies addressing the effects of natural polysaccharides on mitochondrial function or mitochondria-related mechanisms were considered. Reference lists of relevant reviews and primary studies were additionally screened. Studies unrelated to mitochondrial regulation or lacking sufficient information on the investigated polysaccharide were excluded. The selected literature was manually categorized according to structural features, mitochondrial mechanisms, disease applications, and translational relevance.

## 3 Structural basis of NPs for mitochondrial regulation

NPs are structurally heterogeneous carbohydrate macromolecules rather than uniform bioactive compounds. Their mitochondria-related bioactivity is influenced by the structural characteristics, including molecular weight (Mw), monosaccharide composition, glycosidic linkages, branching degree, chemical substitutions, conformation, and charge properties. These parameters may shape mitochondrial outcomes such as ROS production, mitochondrial membrane potential (ΔΨm), OXPHOS, mitophagy, and apoptosis.

### 3.1 Structural diversity of NPs

NPs are derived from plants, fungi, algae, animals, and microorganisms, each providing distinct structural motifs relevant to bioactivity. Plant-derived NPs are commonly enriched in glucans, arabinogalactans, and pectic domains, including homogalacturonan and rhamnogalacturonan-I regions. These structures contain neutral sugars and uronic acids with diverse linkages and branching patterns, which may influence solubility, gut microbial fermentation, antioxidant activity, and mitochondria-related metabolic signaling (Guru et al., 2023). Fungal NPs are typically represented by β-glucans, especially β-1,3-glucan backbones with β-1,6-linked branches. These motifs favor recognition by pattern-recognition receptors such as Dectin-1 and Toll-like receptors, linking fungal polysaccharide structure to immune-metabolic signaling, inflammatory regulation, and mitochondrial responses (Wang et al., 2022). Algal NPs are often characterized by sulfated and uronic acid-rich structures, such as alginate, which consists mainly of β-D-mannuronic acid and α-L-guluronic acid residues. These polyanionic structures may enhance protein binding, membrane interaction, antioxidant activity, and cytoprotection under mitochondrial stress (Liu B. et al., 2025). Animal-derived NPs, including glycosaminoglycans and chitin/chitosan-related polymers, contain repeating disaccharides, amino sugars, uronic acids, and sulfated or acetylated groups, which influence charge density, hydration, matrix interaction, and cell signaling (Yang et al., 2020). In addition to source-dependent structural diversity, NPs also exhibit substantial structural heterogeneity within the same biological source. Geographic origin, cultivation conditions, harvest season, and extraction and purification procedures can alter molecular weight, monosaccharide composition, branching patterns, and other structural characteristics, thereby contributing to variations in bioactivity. Such variability complicates cross-study comparisons and the establishment of reliable structure–activity relationships, highlighting the need for detailed source information and batch-specific structural characterization.

### 3.2 Structural determinants associated with mitochondria-related bioactivity

Mw is an important structural factor influencing the bioavailability and mitochondria-related bioactivity of NPs. Low- or medium-Mw polysaccharide fractions generally show improved solubility, active-group exposure, diffusibility, and cellular uptake potential, whereas high-Mw polysaccharides may act mainly through mucosal retention, receptor clustering, or gut microbiota-mediated pathways (Wang et al., 2023). However, direct evidence linking Mw to defined mitochondrial functional outcomes remains limited. In one comparative study, the low-Mw *Coprinus comatus* polysaccharide fraction CCPJ-80 (∼37 kDa) showed greater mitochondria-related protection than a high-Mw fraction (∼3308 kDa), as reflected by reduced ROS and restored ΔΨm in DSS-injured Caco-2 cells (Chen et al., 2026).

Monosaccharide composition is more commonly associated with upstream bioactivities than with direct mitochondrial endpoints. Fucose-rich or sulfated polysaccharides are often linked to anti-inflammatory effects, uronic acid- and galacturonic acid-rich polysaccharides to antioxidant and antitumor activities, mannose- and galactose-rich polysaccharides to immunomodulatory effects, and neutral sugar-rich polysaccharides to prebiotic activity (Wang et al., 2024). These activities may indirectly influence mitochondrial homeostasis by regulating oxidative stress, inflammatory signaling, microbial metabolites, and immune activation. Some composition-defined preparations have also shown mitochondria-related effects. For example, fucoidan oligosaccharide, a fucose-rich sulfated oligosaccharide, alleviated D-galactose (D-Gal)-induced renal injury partly by preserving mitochondrial function and suppressing excessive mitophagy (Shi et al., 2025). However, this finding does not establish a causal role for fucose content or sulfation alone.

Glycosidic linkage type, branching pattern, and spatial conformation mainly affect receptor recognition and downstream immune-metabolic signaling (Meng et al., 2026). For example, *Dictyophora indusiata* polysaccharide (DIP), characterized as a 1,3-β-D-glucan with 1,4-β- and 1,6-β-D-Glcp branches, showed stronger anti-inflammatory effects against dextran sulfate sodium-induced colitis than *Tremella fuciformis* polysaccharide (TFP), which consisted of a 1,3-α-D-mannose backbone with xylose/fucose side chains (Yuan et al., 2025). Such structural differences may indirectly influence mitochondria-related processes through upstream immune-metabolic signaling, but direct evidence linking specific glycosidic linkages to defined mitochondrial functional outcomes remains limited.

Chemical modifications, including sulfation, carboxymethylation, phosphorylation, and acetylation, can alter charge density, solubility, redox properties, and receptor interactions, thereby modulating NP bioactivity. However, direct links between individual modifications and defined mitochondrial functional outcomes have not been well established. In a related formulation strategy, *Phylloporia fontanesiae* polysaccharide-stabilized selenium nanoparticles enhanced mitochondrial localization and induced ROS-mediated mitochondrial apoptosis in A549 cells, highlighting the potential of selenium-based nano-modification to strengthen mitochondria-directed bioactivity (Ju et al., 2026).

Overall, structural features may shape the bioavailability, molecular interactions, and mitochondria-related bioactivity of NPs, but most reported relationships remain associative rather than causal. Establishing robust structure–activity relationships will require structurally defined comparative series and direct functional mitochondrial validation.

## 4 Multiple mechanisms of NPs against mitochondrial dysfunction

NPs mitigate mitochondrial dysfunction through multiple, interrelated mechanisms rather than through a single molecular target. They mainly restore mitochondrial energy metabolism and structure, regulate mitochondrial quality control, and strengthen cellular defenses against mitochondrial stress. These mechanisms collectively converge on the maintenance of mitochondrial homeostasis and provide the mechanistic basis for the broad therapeutic potential of NPs across different disease settings (Figure 1) (Gu et al., 2024).

**FIGURE 1:**
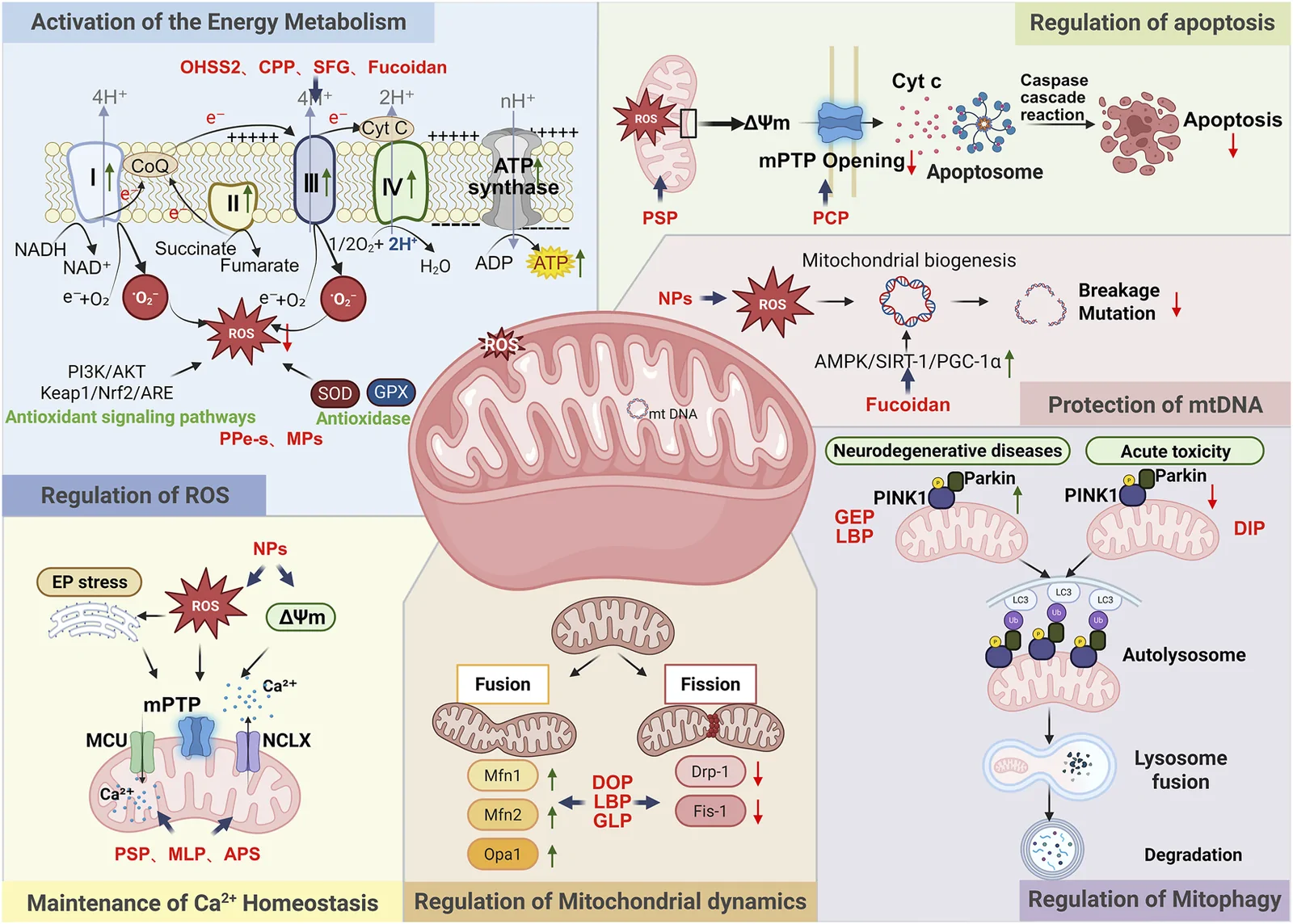
Mechanisms by which NPs regulate mitochondrial homeostasis. Note: OHSS2: *Cladophora oligoclada* sulfated polysaccharide; CPP: *Codonopsis pilosula* polysaccharide; SFG: Sulfated fucogalactan; PPe-s: Pumpkin polysaccharide hydrolysates; MPs: maca polysaccharides; PSP: *Polygonatum sibiricum* polysaccharides; PCP: Pocoa polysaccharides; MLP: Mulberry leaf polysaccharides; APS: *Astragalus* polysaccharides; DOP: *Dendrobium officinale* polysaccharides; LBP: *Lycium barbarum* polysaccharides; GLP: *Ganoderma lucidum* polysaccharides; GEP: *Gastrodia elata* polysaccharide; DIP: *Dictyophora indusiata* polysaccharide. Green upward arrow: Activation/Regulation; Red downward arrow: Inhibition/Reduction. Created in BioRender. (2026) https://www.biorender.com.

### 4.1 Restoration of mitochondrial bioenergetics

Bioenergetic failure is a hallmark of mitochondrial dysfunction. Disruption of the electron transport chain (ETC; complexes I–IV) and OXPHOS impairs electron transfer, reduces ATP generation, and ultimately causes cellular energy stress (Nolfi-Donegan et al., 2020). Recent studies indicate that NPs can alleviate this deficit by maintaining ETC activity, improving OXPHOS efficiency, and promoting mitochondrial biogenesis. For example, the sulfated polysaccharide OHSS2 from *Cladophora oligoclada* enhanced complexes I–III activity, stabilized ΔΨm, and increased ATP production in oxidatively injured NIT-1 cells (He et al., 2022). In a chronic hypoxia mouse model, *Codonopsis pilosula* polysaccharide (CPP) enhanced mitochondrial bioenergetic status by elevating ATP, total adenylate pool, and adenylate energy charge levels and reducing oxidative damage, thereby supporting energy metabolism under hypoxic stress (Li H. et al., 2025). Peroxisome proliferator-activated receptor γ coactivator 1α (PGC-1α) coordinates mitochondrial renewal and energy metabolism, making it a key signaling node in bioenergetic recovery (Halling and Pilegaard, 2020). In line with this, sulfated fucogalactan (SFG) increased the expression of ETC-related genes and proteins through SIRT1/PGC-1α activation in H_2_O_2_-induced β-cell dysfunction, thereby restoring ATP synthesis (Wu et al., 2022). Similarly, fucoidan improved bioenergetic deficits in D-Gal-induced cognitive impairment and ultraviolet-induced skin aging models by activating the AMPK/SIRT1/PGC-1α pathway and promoting mitochondrial renewal (Jing et al., 2021; Xu et al., 2024).

### 4.2 Rebalancing of mitochondrial ROS homeostasis

Mitochondria are a major intracellular source of ROS, especially when respiratory-chain function is impaired (Murphy, 2009). Excessive ROS worsen mitochondrial injury by damaging mtDNA, lipids, and proteins, creating a vicious cycle of oxidative stress and mitochondrial dysfunction (Lu and Gong, 2009; Djouadi and Bastin, 2019; He et al., 2019). NPs may disrupt this cycle by reducing ROS accumulation and enhancing endogenous antioxidant defenses. Studies have shown that NPs can activate redox-related pathways such as PI3K/Akt and Keap1/Nrf2/ARE, thereby strengthening cellular antioxidant capacity (Chen N. et al., 2024; Chen X. et al., 2025). For example, pumpkin polysaccharide hydrolysates (PPe-s) increased the activities of superoxide dismutase (SOD) and glutathione peroxidase (GSH-Px) (Gao et al., 2023), whereas maca polysaccharides (MPs) activated the hepatic Nrf2/GPX/SOD antioxidant axis to strengthen endogenous antioxidant capacity (Zhang J. et al., 2024). These findings indicate that NPs help preserve mitochondrial homeostasis by rebalancing intracellular redox status.

### 4.3 Regulation of mitochondria-dependent apoptosis

Mitochondria-dependent apoptosis is a key consequence of mitochondrial damage under oxidative or toxic stress. Disruption of mitochondrial membranes triggers ΔΨm collapse, sustained opening of the mitochondrial permeability transition pore (mPTP), and release of cytochrome c (Cyt c) into the cytoplasm. This event activates downstream apoptotic proteins such as Caspase-9 and Caspase-3, ultimately leading to programmed cell death (Chipuk and Green, 2008; Lalier et al., 2022). NPs can counteract this apoptotic cascade by stabilizing ΔΨm, inhibiting pathological mPTP opening, and reducing ROS accumulation. For instance, *Polygonatum sibiricum* polysaccharides (PSP) restored ΔΨm and reduced ROS levels in C2C12 cells via activation of the PI3K/Akt/mTOR pathway, thereby suppressing mitochondria-dependent apoptosis (Li Y. et al., 2025). In addition, Pocoa polysaccharides (PCP) protected HK-2 cells from calcium oxalate-induced oxidative stress by preventing sustained mPTP opening and attenuating Cyt c release, which alleviated caspase-mediated apoptotic injury (Xiong et al., 2023). These findings suggest that NPs help prevent the progression from mitochondrial stress to cell death.

### 4.4 Regulation of mitophagy

Mitophagy is a crucial mitochondrial quality-control mechanism that selectively eliminates damaged mitochondria to maintain cellular homeostasis. Dysregulation of key mitophagy regulators, such as PTEN-induced putative kinase 1 (PINK1) and Parkin, impairs mitochondrial clearance, leading to mitochondrial dysfunction and cellular stress (Lu et al., 2018; Picca et al., 2023). Conversely, excessive mitophagy under acute stress can also exacerbate cellular injury. Thus, maintaining an optimal level of mitophagic activity is essential for mitochondrial integrity. NPs appear to regulate mitophagy in a context-dependent manner. Under conditions of impaired mitochondrial clearance, they can enhance mitophagy and promote mitochondrial renewal. For example, *Gastrodia elata* polysaccharide (GEP) activated the PINK1/Parkin pathway in 1-methyl-4-phenyl-1,2,3,6-tetrahydropyridine-induced Parkinson’s mouse model, thereby enhancing mitophagy and reducing apoptotic injury (Li S. et al., 2025). *Lycium barbarum* polysaccharides (LBP) similarly stimulated AMPK/PINK1/Parkin-mediated mitophagy in obese mice, improving glucolipid metabolism and skeletal muscle mitochondrial function (Ren et al., 2025). By contrast, in arsenic-induced hepatotoxicity, DIP suppressed excessive PINK1/Parkin-dependent mitophagy and reduced intracellular ROS to mitigate liver injury (Hu et al., 2022). These findings highlight that NPs maintain mitochondrial homeostasis by finely tuning mitophagy in accordance with the specific pathological context.

### 4.5 Protection of mitochondrial DNA integrity

Mitochondrial DNA (mtDNA) is crucial for OXPHOS and mitochondrial homeostasis. Due to its proximity to the ETC, lack of histone protection, and limited repair capacity, mtDNA is highly vulnerable to oxidative damage, which can further impair mitochondrial function (Grazioli and Pugin, 2018). NPs appear to protect mtDNA mainly by mitigating oxidative stress and stimulating mitochondrial biogenesis. For instance, fucoidan prevented ethanol-induced reductions in mtDNA copy number in rat hepatocytes and enhanced ATP production (Zhao et al., 2021). In ultraviolet-induced HaCaT cells, fucoidan activated the AMPK/SIRT1/PGC-1α pathway, promoted mitochondrial biogenesis, and reduced ROS-mediated mtDNA damage (Jing et al., 2021). These results suggest that NPs help maintain mitochondrial genetic stability, thereby supporting overall mitochondrial function.

### 4.6 Restoration of mitochondrial dynamic balance

Mitochondrial dynamics are governed by a tightly regulated balance between fission and fusion, which is essential for maintaining mitochondrial homeostasis. Fusion, mediated by Mfn1, Mfn2, and OPA1, preserves mitochondrial integrity and enables content exchange, while fission, driven by p-Drp1 and Fis1, removes damaged regions for quality control. Under pathological conditions, excessive fission or insufficient fusion disrupts mitochondrial network architecture and impairs mitochondrial function (Cho and Kim, 2024). NPs appear to counteract this imbalance by suppressing pathological fission and supporting physiological fusion. *Dendrobium officinale* polysaccharides (DOP) alleviated thiacloprid-induced cardiotoxicity by rebalancing the expression of mitochondrial fission- and fusion-related proteins (Han et al., 2025). LBP reduced hyperglycemia-aggravated ischemic brain injury in rats by inhibiting p-Drp1 upregulation and preventing OPA1 downregulation (Liu et al., 2017). Likewise, *Ganoderma lucidum* polysaccharides (GLP) decreased p-Drp1 and Fis1 expression while increasing Mfn1, Mfn2, and OPA1 levels, thereby attenuating pathological mitochondrial fragmentation in mice (Liu X. et al., 2025). These findings indicate that NPs help maintain mitochondrial morphology and function by rebalancing the fission–fusion network.

### 4.7 Maintenance of mitochondrial calcium homeostasis

Mitochondrial calcium (mtCa^2+^) homeostasis is essential for mitochondrial function and cellular survival. Impaired mtCa^2+^ handling can cause calcium overload, reduce ATP production, increase oxidative stress, and activate cell death pathways (Giacomello et al., 2007; De Ridder et al., 2023). NPs help preserve mtCa^2+^ balance by regulating calcium transport and preventing pathological calcium accumulation. For example, PSP attenuated D-Gal-induced Ca^2+^ elevation in C2C12 myoblasts by modulating calcium-transport-related proteins (Chen W. et al., 2024). Mulberry leaf polysaccharides (MLP) similarly reversed H_2_O_2_-induced mtCa^2+^ overload in peripheral blood leukocytes by suppressing the expression of calcium transport-related genes (Jiang et al., 2024). In high-glucose (HG)-treated rat aortic endothelial cells, *Astragalus* polysaccharides (APS) upregulated the mitochondrial Na^+^/Ca^2+^ exchanger NCLX, thereby supporting mtCa^2+^ homeostasis (Miao et al., 2022). These findings suggest that NPs help preserve mitochondrial bioenergetic function by stabilizing mitochondrial calcium handling.

## 5 Therapeutic applications of NPs regulating mitochondrial dysfunction in diseases

Based on the mechanisms discussed above, this section summarizes the therapeutic applications of NPs in representative disease models, including neurodegenerative, metabolic, cardiovascular, malignant, and liver diseases. Across disease categories, mechanistic confidence is greater when mitochondrial effects are supported by functional assays rather than by indirect redox, apoptotic, or pathway markers alone. Because polysaccharide preparations vary in structure and experimental conditions, differences across studies should not be attributed to specific structural features without direct comparative evidence. Notably, NPs exhibit context-dependent mitochondrial regulatory effects. In non-malignant diseases, NPs preserve mitochondrial homeostasis by inhibiting oxidative stress and apoptosis, thereby protecting normal cells. In cancer, however, NPs can intentionally disrupt mitochondrial function by inducing ROS overload and activating apoptosis, thereby suppressing malignant cells. Figure 2 summarizes the major mitochondrial targets of NPs and their therapeutic implications across different disease contexts.

**FIGURE 2:**
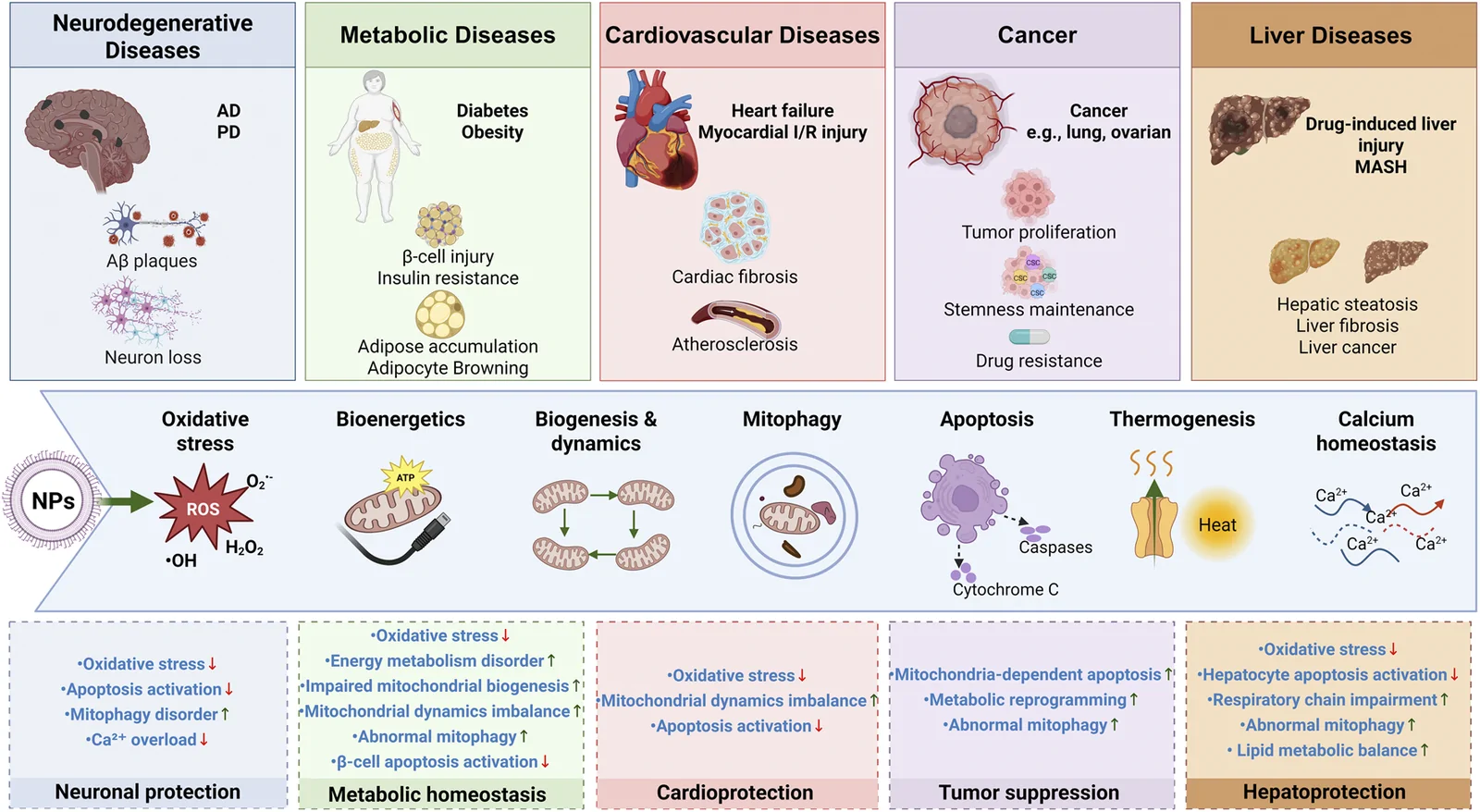
Disease-context-dependent mitochondrial effects of NPs. Note: Green upward arrow: Activation/Regulation; Red downward arrow: Inhibition/Reduction. Created in BioRender. (2026) https://www.biorender.com.

### 5.1 Neurodegenerative diseases

Neurodegenerative diseases, particularly Alzheimer’s disease (AD) and Parkinson’s disease (PD), are closely linked to mitochondrial integrity because neurons depend on ATP production to maintain synaptic activity and other energy-demanding functions. Although the brain contains fewer mitochondria than skeletal muscle, it consumes a disproportionately large amount of oxygen, making neurons especially vulnerable to mitochondrial dysfunction. Impaired OXPHOS, excessive mitochondrial ROS generation, defective mitophagy, calcium dyshomeostasis, and mitochondria-dependent apoptosis are closely associated with amyloid-β (Aβ) accumulation, tau pathology, α-synuclein toxicity, dopaminergic neuronal loss, and progressive cognitive or motor decline (Shoshan-Barmatz et al., 2018; Zhang W. et al., 2023). Therefore, this section discusses how NPs regulate these mitochondrial abnormalities to support neuronal survival in neurodegenerative diseases (Table 1).

**TABLE 1:** NPs regulating mitochondrial dysfunction in neurodegenerative diseases.

Name	Source	Mw/Monosaccharide composition	Disease	Animals/Cells	Mitochondrial function	Mechanism/efficacy	Ref
Fucoidan	Brown algae	266.1 kDa/L-Fuc	Aβ(25-35)/D-Gal-induced AD model	Male ICR mice /PC12 cells	Oxidative stress Mitochondrial apoptosis	Fucoidan → ↑ SOD/GSH + ↓ Cyt c–caspase-9/3 apoptosis → improved Aβ/D-Gal-induced neurotoxicity	(Wei et al., 2017)
Fucoidan	Brown alga	7 kDa/Fuc, Gal	Rotenone-induced PD model	Male SD rats/NG	Oxidative stress Mitochondrial energy metabolism	Fucoidan → ↑ PGC-1α/NRF2 → restored mitochondrial respiration and complex I/II activity → protected dopaminergic neurons	(Zhang et al., 2018)
ACPS	Fruiting bodies of *Amanita caesarea*	18.62 kDa and 33.5 kDa/Xyl, Man, Gal, Glc	D-Gal/AlCl_3_-induced AD model	Male BALB/c mice/HT22 cells	Oxidative stress Mitochondrial apoptosis	ACPS → ↑ Nrf2/HO-1 → ↓ ROS/Ca^2+^ overload + restored ΔΨm → ↓ Cyt c/Bax/caspase-3 apoptosis → improved AD-like neuronal injury	(Li Z. et al., 2019)
CYP	*Corydalis yanhusuo*	75.5 kDa/D-Glc	Aβ-induced AD model	NG/PC12 cells	Mitochondrial apoptosis	CYP → restored ΔΨm → ↓ Cyt c release and caspase-9/3 activation → reduced Aβ-induced apoptosis	(He et al., 2020)
CCP	*Coptis chinensis* Franch	3.96 kDa/Glc	Aβ-induced AD model	NG/PC12 cells	Mitochondrial apoptosis	CCP → ↓ JNK phosphorylation → restored ΔΨm + ↓ Cyt c/caspase-3 signaling → reduced Aβ-induced apoptosis	(Li Y. et al., 2019)
ACP	*Antrodia camphorata*	NG/NG	6-OHDA-induced PD model	Mice(strain not reported)/MES23.5 cells	Oxidative stress Mitochondrial apoptosis	ACP → ↓ ROS–NLRP3 activation → ↓ neuroinflammation and dopaminergic neuronal apoptosis → improved PD-like dysfunction	(Han et al., 2020)
GP4	*Panax ginseng* C. A Meyer	4.7 kDa/Glc, GalA, Gal, Arab, Rha, GlcA, Man	Aβ-induced AD model	Transgenic *C. elegans*/SH-SY5Y cells	Mitophagy	GP4 → ↑ PINK1/Parkin-mediated mitophagy → improved mitochondrial clearance → ↓ Aβ accumulation → improved AD-like aging	(Zhang S. et al., 2023)
IPS1 and IPS2	Mycelium of *Paecilomyces cicadae* TJJ1213	IPS1: 2400 kDa, IPS2: 679 kDa/IPS1: Man,Glc,Gal; IPS2: Man,Glc,Gal	H_2_O_2_-induced oxidative neuronal injury	NG/PC12 cells	Oxidative stress Mitochondrial apoptosis Mitophagy	IPS1/IPS2 → ↓ ROS/Ca^2+^ overload + restored ΔΨm → ↓ mitochondrial apoptosis and excessive mitophagy → protection against oxidative neuronal injury	(Tian et al., 2023)

ACPS: *amanita caesarea* polysaccharides; CYP: *corydalis yanhusuo* polysaccharide; CCP: *coptis chinensis* polysaccharide; ACP: *antrodia camphorata* polysaccharide; GP4: ginseng polysaccharide; IPS1 and IPS2: Polysaccharides from *Paecilomyces cicadae* TJJ1213. Fuc, fucose; Gal, galactose; Glc, glucose; Xyl, xylose; Man, mannose; Rha, rhamnose; Arab, arabinose; GlcA, glucuronic acid; GalA, galacturonic acid. “NG”: not given; “↑”: increase or upregulation; “↓”: decrease or downregulation.

In AD- and PD-related models, NPs mainly exert neuroprotective effects by reducing mitochondrial oxidative stress and enhancing endogenous antioxidant defenses. Fucoidan is a representative example. It attenuated Aβ-induced oxidative injury and cholinergic dysfunction in an AD model and improved mitochondrial antioxidant capacity and respiratory function in a rotenone-induced PD model by activating the PGC-1α/Nrf2 pathway (Wei et al., 2017; Zhang et al., 2018). This pathway is highly relevant to neurodegeneration because it links Nrf2-mediated antioxidant defense with PGC-1α-dependent mitochondrial renewal, thereby strengthening neuronal resistance to chronic oxidative stress. Another example is *Amanita caesarea* polysaccharides (ACPS), a relatively low-Mw heteropolysaccharide fraction. ACPS showed neuroprotective effects in both L-glutamic acid-injured HT22 cells and a D-Gal/aluminum trichloride-induced AD mouse model by activating Nrf2-mediated antioxidant defense, preserving mitochondrial function, reducing Aβ deposition, and improving oxidative stress and cholinergic dysfunction (Li Z. et al., 2019).

Mitochondria-dependent apoptosis is another key pathological process in AD and PD. Aβ deposition, tau aggregation, and neurotoxins can induce mitochondria-dependent apoptosis, ultimately contributing to neuronal loss (Ashleigh et al., 2023). Several NPs have been reported to interrupt this process. A purified polysaccharide from *Corydalis yanhusuo* (CYP) protected PC12 cells against Aβ(25–35)-induced apoptosis by preserving ΔΨm, reducing Cyt c release, restoring Bax/Bcl-2 balance, and suppressing caspase activation (He et al., 2020). Similarly, *Coptis chinensis* polysaccharide (CCP) attenuated Aβ(25–35)-induced apoptosis in PC12 cells by preserving mitochondrial membrane potential, reducing Cyt c release, and suppressing JNK-mediated Bax/Bcl-2 imbalance and caspase-3 activation (Li Y. et al., 2019). In a PD-related model, *Antrodia camphorata* polysaccharide (ACP) attenuated 6-hydroxydopamine (6-OHDA)-induced dopaminergic neuronal injury by suppressing ROS-NLRP3 inflammasome activation, thereby reducing apoptosis and improving motor dysfunction in PD mice (Han et al., 2020).

Defective mitophagy contributes to neurodegenerative progression by allowing damaged mitochondria to accumulate. NP-mediated regulation of mitophagy appears to be context-dependent. In a *Caenorhabditis elegans* AD model, a 4.7-kDa ginseng polysaccharide (GP4) reduced Aβ accumulation, neuroinflammation, and memory deficits by promoting neuronal mitophagy (Zhang S. et al., 2023). Conversely, intracellular polysaccharides from *Paecilomyces cicadae* TJJ1213 (IPS1 and IPS2) protected H_2_O_2_-injured PC12 cells by suppressing excessive PINK1/Parkin-mediated mitophagy, together with reducing ROS generation, Ca^2+^ leakage, and apoptotic injury (Tian et al., 2023).

Overall, NPs show neuroprotective potential in AD- and PD-related models through modulation of mitochondrial oxidative stress, apoptosis, and quality control. Divergent effects on mitophagy likely reflect differences in baseline mitochondrial status, disease model, stress severity, and treatment conditions rather than a uniform pathway response. Because the investigated polysaccharides also differ in molecular weight, composition, and purity, these differences cannot be readily attributed to specific structural features. Whether NPs cross the blood–brain barrier or directly engage neuronal mitochondrial targets remains insufficiently established.

### 5.2 Metabolic diseases

Metabolic diseases, including diabetes, obesity, non-alcoholic fatty liver disease (NAFLD), and diabetic complications, are closely associated with mitochondrial dysfunction. In metabolically active tissues such as pancreatic β-cells and adipose tissue, mitochondria are central to metabolic homeostasis by coordinating energy production, substrate metabolism, redox control, and inflammatory responses. Mitochondrial dysfunction disrupts these processes, thereby promoting metabolic inflammation, insulin resistance, β-cell failure, and diabetes-related tissue injury (Cojocaru et al., 2023). Therefore, this section discusses how NPs modulate these mitochondrial abnormalities in metabolic diseases (Figure 3).

**FIGURE 3:**
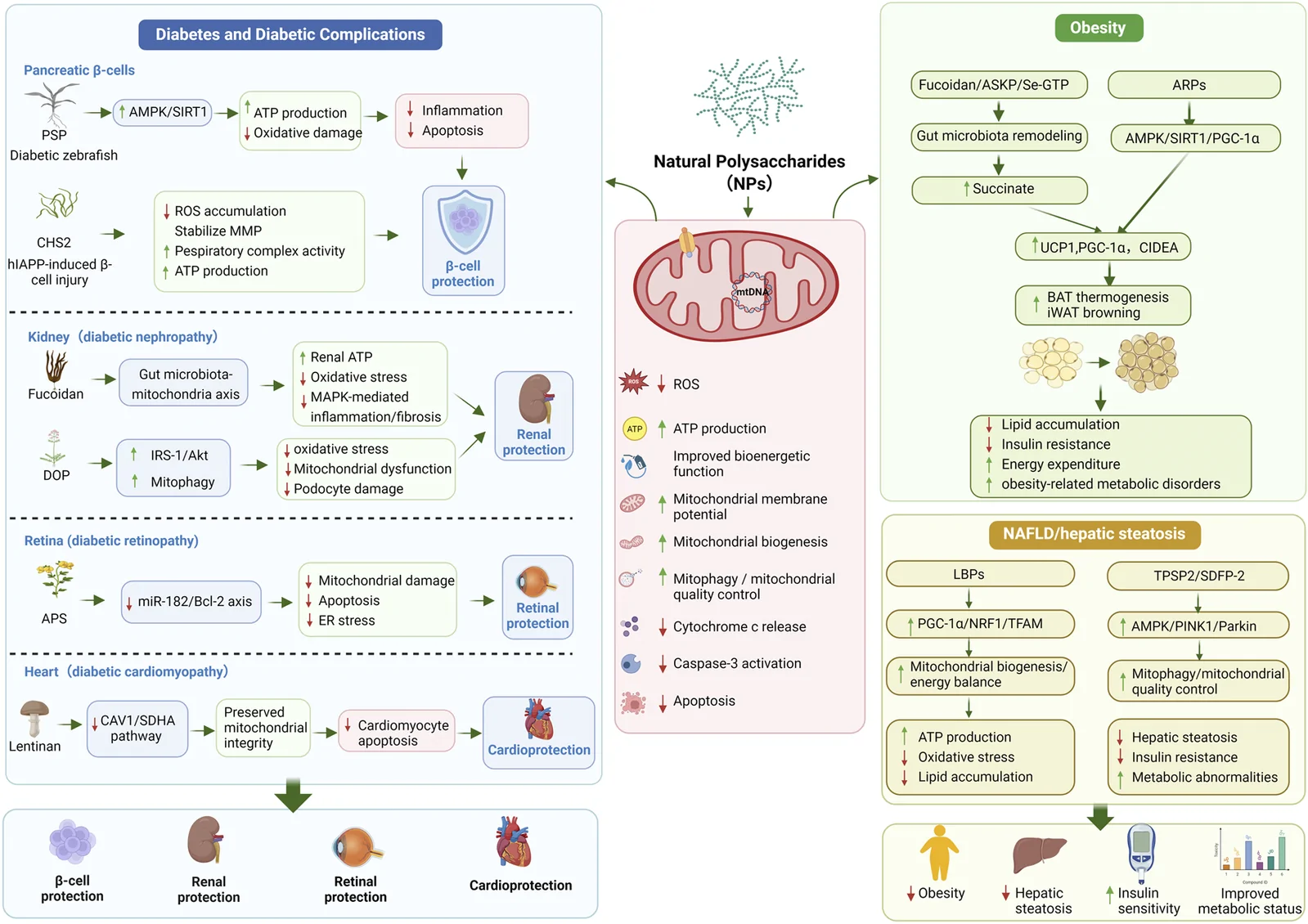
Mitochondrial regulation by NPs in metabolic diseases. Note: PSP: *Polygonatum sibiricum* polysaccharides; CHS2: A sulfated polysaccharide from *Chaetomorpha linum*; DOP: *Dendrobium officinale* polysaccharides; APS: *Astragalus* polysaccharides; ARPs: *Anoectochilus roxburghii* polysaccharides; LBP: *Lycium barbarum* polysaccharides; TPSP2: *Purified polysaccharide TPSP2 from Tremella fuciformis*; SDFP-2: Dragon fruit polysaccharide. Green upward arrow: Activation/Regulation; Red downward arrow: Inhibition/Reduction. Created in BioRender. (2026) https://www.biorender.com.

In diabetes and its complications, NPs mainly protect metabolically vulnerable tissues by reducing mitochondrial oxidative stress, preserving bioenergetic function, and inhibiting mitochondria-dependent apoptosis. PSP activated the AMPK/SIRT1 pathway to relieve mitochondrial oxidative injury, inhibit inflammation and pancreatic β-cell apoptosis, thereby improving β-cell function and exerting hypoglycemic effects in diabetic zebrafish (Lin et al., 2025). CHS2, a sulfated polysaccharide from *Chaetomorpha linum*, further protected NIT-1 β-cells against human islet amyloid polypeptide (hIAPP)-induced injury by reducing ROS accumulation while preserving ΔΨm, respiratory complex activity, and ATP production (Qin et al., 2022). Beyond β-cell protection, NPs also alleviate mitochondrial injury in diabetic target organs. In early diabetic nephropathy, fucoidan improved renal dysfunction by modulating the gut microbiota–mitochondria axis, increasing renal ATP levels, alleviating mitochondrial oxidative stress, and suppressing MAPK-mediated inflammation and fibrosis (Zhong et al., 2024). DOP further alleviated HG-induced podocyte injury in diabetic nephropathy by regulating IRS-1/Akt signaling and promoting mitophagy-associated mitochondrial quality control (Li H. et al., 2023). In diabetic retinopathy, APS attenuated HG-induced mitochondrial damage and apoptosis in retinal cells by modulating the miR-182/Bcl-2 axis (Gao et al., 2021). Lentinan alleviated diabetic cardiomyopathy in db/db mice by suppressing the CAV1/SDHA pathway, thereby preserving mitochondrial integrity and reducing cardiomyocyte apoptosis (Hu S. et al., 2023).

In obesity, mitochondrial dysfunction contributes to metabolic stress by impairing energy expenditure, promoting adipose inflammation, and facilitating lipid accumulation (Tang et al., 2023). Several NPs have been reported to counteract obesity-related metabolic dysfunction by enhancing mitochondria-associated adipose thermogenesis. In high-fat diet (HFD)-induced obese mice, *Artemisia sphaerocephala* Krasch polysaccharide (ASKP) and selenium-enriched green tea polysaccharides (Se-GTP) promoted brown adipose tissue (BAT) thermogenesis and inguinal white adipose tissue (iWAT) browning, partly through gut microbiota remodeling, increased succinate production, and increased protein expression of UCP1 and PGC-1α (Zeng et al., 2022; Li D. et al., 2023). *Anoectochilus roxburghii* polysaccharides (ARPs) also protected mice against diet-induced obesity by activating the AMPK/SIRT1/PGC-1α pathway, inducing thermogenic gene expression in adipose tissue, and reducing hepatic lipid accumulation (Tian et al., 2022).

NPs also modulate hepatic mitochondrial function in metabolic diseases, particularly NAFLD and obesity-associated hepatic steatosis. In NAFLD-related models, LBP activated the PGC-1α/NRF1/TFAM pathway to promote mitochondrial biogenesis, accompanied by increased ATP production, reduced oxidative stress, and decreased hepatic lipid accumulation (Zhang Y. N. et al., 2022). In HFD-induced metabolic disorders, *T. fuciformis* polysaccharide TPSP2 (Lu et al., 2025a) and dragon fruit polysaccharide SDFP-2 (Lu et al., 2025b) restored hepatic mitochondrial quality control by activating AMPK/PINK1/Parkin-mediated mitophagy, thereby improving hepatic steatosis, insulin resistance, and related metabolic abnormalities.

Overall, NPs may improve metabolic disorders through tissue-specific regulation of mitochondrial homeostasis, but the underlying mechanisms vary across models. Some studies provide direct functional evidence involving respiratory-chain activity or ATP production, whereas others suggest indirect effects mediated through gut microbiota remodeling and microbial metabolites. These distinct pathways should not be mechanistically conflated, and differences across studies cannot be readily attributed to specific polysaccharide structural features.

### 5.3 Cardiovascular diseases

Cardiovascular diseases (CVD) remain a major cause of morbidity and mortality worldwide, particularly in aging populations (GBD, 2019 Diseases and Injuries Collaborators, 2020). Cardiomyocytes and vascular endothelial cells are highly dependent on mitochondrial function, as mitochondrial energy production and redox–calcium homeostasis are essential for myocardial contraction and vascular integrity. Under pathological conditions, including myocardial ischemia/reperfusion (I/R), diabetic vascular injury, drug-induced cardiotoxicity, and cardiac aging, mitochondrial dysfunction drives energetic failure, oxidative stress, mitochondrial quality-control defects, and mitochondria-dependent apoptosis (Diaz-Vegas et al., 2020; Yang et al., 2025). This section therefore discusses how NPs protect the cardiovascular system by restoring mitochondrial homeostasis in representative CVD models (Table 2).

**TABLE 2:** NPs regulating mitochondrial dysfunction in cardiovascular diseases.

Name	Source	Mw/Monosaccharide composition	Disease	Animals/Cells	Mitochondrial function	Mechanism/efficacy	Ref
Fucoidan	Brown algae	NG/NG	DOX-induced cardiotoxicity	Male C57BL/6J mice/NG	Oxidative stress Mitochondrial energy metabolism	Fucoidan → ↓ ROS + ↑ SOD/GSH-Px → improved mitochondrial respiratory chain function and ATP production → alleviated cardiotoxicity	(Ji et al., 2022)
PSG	*Ganoderma atrum*	1013 kDa/Glc, Man, Gal	DOX-induced cardiotoxicity	Kunming mice/Isolated cardiac myocytes	Mitochondrial apoptosis Oxidative stress	PSG → ↓ mitochondrial ROS + ↑ MnSOD → restored ΔΨm and inhibited mPTP opening → ↓ Cyt c–caspase apoptosis → alleviated cardiotoxicity	(Li et al., 2018)
PSG	*Ganoderma atrum*	1013 kDa/Glc, Man, Gal	PD-1 inhibitor-induced carditis	Female BALB/c mice/NG	Mitochondrial dynamics Oxidative stress	PSG → restored mitochondrial dynamics, ↑ Drp1 and ↓ Mfn2 → maintained ΔΨm and ↓ mitochondrial ROS → ↓ NLRP3-mediated pyroptosis → alleviated carditis	(Mu et al., 2025)
LBP	*Lycium barbarum*	31.9 kDa/Arab, Glc, Gal	Myocardial I/R Injury	SD rat/H9C2 cardiomyoblasts	Mitochondrial apoptosis Mitochondrial dynamics Oxidative stress	LBP → ↓ GRK2 signaling → restored mitochondrial fission/fusion balance and AKT/eNOS signaling → ↓ ROS and cardiomyocyte apoptosis → alleviated myocardial I/R injury	(Li et al., 2022)
DSP	*Salvia miltiorrhiza* Bunge	NG/NG	Cerebral I/R Injury	Male Wistar rats/NG	Oxidative stress	DSP → ↓ mitochondrial ROS + ↑ mitochondrial SOD/CAT/GSH-Px → ↓ oxidative brain injury → alleviated cerebral I/R injury	(Tu et al., 2013)
APS	*Astragalus* membranaceus	40–80 kDa/Glc, Arab, Gal, Rha, GalA	High glucose-induced diabetic cardiomyopathy	NG/H9C2 cells	Mitochondrial apoptosis	APS → ↓ Cyt c release + ↓ caspase-9/3 + ↑ Bcl-2/Bax ratio → inhibited HG-induced H9C2 apoptosis	(Sun et al., 2017)
PCCL	*Cuscuta chinensis* Lam	NG/NG	D-Gal-induced cardiac aging	Male SD rats/isolated rat cardiomyocytes	Mitochondrial apoptosis	PCCL → ↓ Ca^2+^ overload and Cyt c release → ↓ caspase-3 activation + ↑ Bcl-2/Bax ratio → reduced cardiomyocyte apoptosis	(Sun et al., 2014)
MCP	*Momordica charantia*	NG/NG	Isoproterenol-induced myocardial infarction	Male Wistar rats/NG	Oxidative stress Mitochondrial apoptosis	MCP → inhibition of NF-κB signaling → ↓ oxidative stress and inflammation → ↓ Bax/caspase-3 + ↑ Bcl-2 → alleviated myocardial infarction	(Raish, 2017)

PSG: *ganoderma atrum* polysaccharide; LBP: *lycium barbarum* polysaccharides; DSP: danshen polysaccharides; APS: *astragalus* polysaccharides; PCCL: *cuscuta chinensis* lam polysaccharide; MCP: *Momordica charantia* polysaccharide. Fuc, fucose; Gal, galactose; Glc, glucose; Xyl, xylose; Man, mannose; Rha, rhamnose; Arab, arabinose; GlcA, glucuronic acid; GalA, galacturonic acid. “NG”: not given; “↑”: increase or upregulation; “↓”: decrease or downregulation.

In therapy-related cardiotoxicity models, NPs protect cardiac tissues by restoring mitochondrial homeostasis. Fucoidan alleviated doxorubicin (DOX)-induced cardiotoxicity by improving respiratory-chain function, increasing ATP production, and reducing ROS and lipid peroxidation (Ji et al., 2022). *Ganoderma atrum* polysaccharide (PSG) protected against DOX-induced cardiotoxicity in mice by maintaining mitochondrial redox homeostasis and inhibiting the mitochondria-dependent apoptotic pathway (Li et al., 2018). Notably, PSG attenuated PD-1 inhibitor-induced carditis in Lewis lung carcinoma mice, mainly by restoring mitochondrial dynamic balance, reducing ROS stress, and suppressing NLRP3/Caspase-1/GSDMD-mediated pyroptosis (Mu et al., 2025).

NPs also exert protective effects against I/R-related cardiovascular injury and vascular dysfunction, characterized by mitochondrial and endothelial dysfunction, including excessive ROS generation, impaired nitric oxide signaling, and disrupted fission–fusion homeostasis. In a rat myocardial I/R injury model, LBP reduced infarct damage and cardiomyocyte apoptosis, partly by restoring mitochondrial fission–fusion balance and activating AKT/eNOS signaling, suggesting a role in both cardiomyocyte mitochondrial preservation and endothelial functional recovery (Li et al., 2022). In a rat cerebral I/R model, Danshen polysaccharides (DSP) reduced mitochondrial ROS accumulation and reinforced endogenous antioxidant defenses, supporting their potential protective effects against ischemia-related vascular oxidative injury (Tu et al., 2013).

In cardiomyocyte injury associated with metabolic stress, aging, or other stressors, NPs appear to attenuate mitochondria-dependent apoptosis and calcium-related mitochondrial dysfunction. APS reduced HG-induced apoptosis in H9C2 cardiomyocytes by increasing the Bcl-2/Bax ratio and limiting Cyt c release (Sun et al., 2017). In a D-galactose-induced aging rat model, *Cuscuta chinensis* Lam polysaccharide (PCCL) directly targeted the mitochondrial apoptosis pathway, as shown by reduced intracellular Ca^2+^ overload, Cyt c release, caspase-3 activity, and restoration of the Bcl-2/Bax ratio (Sun et al., 2014). In isoproterenol-induced myocardial infarction model, *Momordica charantia* polysaccharide (MCP) enhanced antioxidant enzyme activity, reduced lipid peroxidation, and suppressed apoptosis by downregulating caspase-3 and improving the Bcl-2/Bax balance (Raish, 2017).

Overall, NPs show cardioprotective potential across cardiotoxicity, ischemic, metabolic, and aging-related models, but the underlying mitochondrial mechanisms vary among pathological settings and cardiovascular cell types. Differences among cardiomyocyte- and endothelial-based models limit direct comparison across studies and make structure–function relationships difficult to infer.

### 5.4 Cancer

Cancer remains a major global health challenge. Despite advances in targeted therapy and immunotherapy, treatment failure remains common because of tumor heterogeneity, recurrence, and drug resistance (Siegel et al., 2022). Mitochondria are central to tumor biology because they integrate energy metabolism, redox control, and cell-death signaling, thereby supporting malignant adaptation and therapeutic tolerance (Liu et al., 2023). In cancer cells, key mitochondrial vulnerabilities include reliance on metabolic and redox flexibility, resistance to mitochondria-dependent apoptosis, and mitophagy-mediated survival under therapeutic stress. Therefore, this section discusses how NPs target mitochondrial stress-response programs that govern tumor cell metabolism, survival, and quality control in cancer models (Figure 4).

**FIGURE 4:**
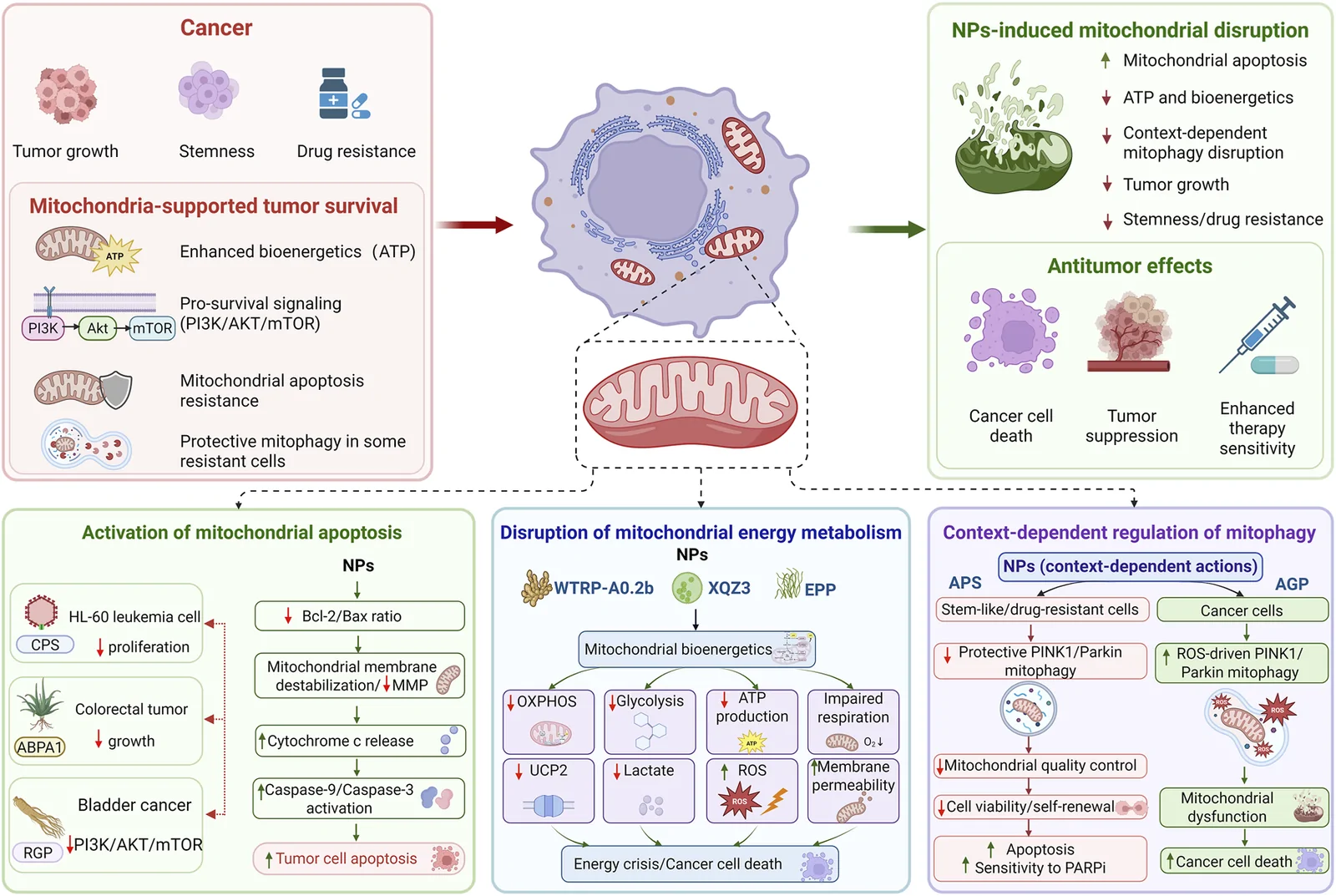
Mitochondrial regulation by NPs in cancer. Note: APS: *Astragalus* polysaccharides; AGP: *Aloe* gel glucomannan; WTRP-A0.2b: Pectic polysaccharide from *Typhonii* Rhizoma; XQZ3: Acidic polysaccharide from *Chlorella pyrenoidosa*; EPP: *Enteromorpha prolifera* polysaccharide; CPS: Capsular polysaccharides; ABPA1: Acetylated mannan from *Aloe vera*; RGP: Red ginseng polysaccharide. Green upward arrow: Activation/Regulation; Red downward arrow: Inhibition/Reduction. Created in BioRender. (2026) https://www.biorender.com.

A major anticancer mechanism of NPs is the induction of mitochondrial apoptosis. In many tumors, suppression of mitochondrial apoptosis enables malignant cells to survive under stress and contributes to tumor persistence, progression, and therapeutic resistance. For example, a capsular polysaccharide (CPS) from the marine bacterium *Cobetia marina* induced apoptosis of HL-60 leukemia cells through mitochondrial membrane potential collapse, ROS accumulation, a reduced Bcl-2/Bax ratio, and activation of initiator and executioner caspases (Kokoulin et al., 2025). An acetylated mannan isolated from *Aloe vera* (ABPA1) suppressed colorectal tumor growth by increasing mitochondrial membrane permeability, promoting Bax translocation, inducing Cyt c release, and activating the caspase cascade (Tong et al., 2022). In bladder cancer models, red ginseng polysaccharide (RGP) inhibited PI3K/AKT/mTOR-related survival signaling and promoted BCL-2 family-dependent mitochondrial dysfunction and apoptosis (Ullah et al., 2026).

Mitochondrial energy metabolism represents another cancer-specific vulnerability, as metabolic flexibility between glycolysis and OXPHOS enables tumor cells to maintain bioenergetic adaptation under stress (Ahn and Metallo, 2015). By disrupting this adaptability, NPs can increase bioenergetic stress and promote tumor cell death. For example, WTRP-A0.2b, a 43-kDa pectic polysaccharide from *Typhonii* Rhizoma containing RG-I, RG-II, and homogalacturonan domains, inhibited K562 cell proliferation by inducing mitochondrial damage and suppressing glycolysis. These effects were accompanied by ROS accumulation, altered oxygen consumption, reduced UCP2 protein expression, and decreased lactate production (Qi et al., 2025). XQZ3, a 29.13-kDa acidic branched polysaccharide from *Chlorella pyrenoidosa*, suppressed tumor growth in patient-derived organoid and xenograft models by inhibiting the HSP90/AKT/mTOR signaling cascade, thereby impairing mitochondrial bioenergetics and promoting mitochondrial dysfunction, autophagy, and apoptosis (Sun et al., 2024). In addition, combined treatment with *Enteromorpha prolifera* polysaccharide (EPP) and DOX reduced ATP production, impaired mitochondrial respiratory function, and enhanced apoptosis in A549 cells, suggesting that NPs may improve chemotherapy efficacy by weakening tumor mitochondrial energy metabolism (Li et al., 2024a).

Mitophagy regulation by NPs appears to be highly context-dependent in cancer. In stem-like or drug-resistant tumor cells, where mitophagy supports mitochondrial quality control and survival under therapeutic stress, its inhibition can weaken tumor cell adaptability. For example, APS acted synergistically with poly(ADP-ribose) polymerase inhibitors to suppress PINK1/Parkin-mediated mitophagy in ovarian cancer stem cells, thereby reducing viability and self-renewal capacity and promoting apoptosis (Peng et al., 2024). Conversely, in some tumor contexts, excessive mitophagy may exceed the adaptive capacity of cancer cells and drive mitochondrial collapse. *Aloe* gel glucomannan (AGP) suppressed CT26 colon cancer cell growth by inducing ROS-driven, PINK1/Parkin-mediated excessive mitophagy, ultimately leading to tumor cell death (Zhang K. et al., 2022).

Overall, NPs may exert anticancer effects by disrupting mitochondrial stress adaptation in a context-dependent manner. Both inhibition of adaptive mitophagy and induction of excessive mitophagy can promote tumor-cell death, depending on the mitochondrial state of the tumor. However, selective mitochondrial effects of NPs on malignant versus normal cells have not yet been clearly established, as most studies lack direct comparisons with normal cells or tissues.

### 5.5 Liver diseases

The liver is highly dependent on mitochondrial integrity because hepatic mitochondria coordinate energy metabolism, redox homeostasis, detoxification, and cell survival. Across liver diseases, mitochondrial dysfunction is commonly characterized by oxidative stress, impaired quality control, disrupted lipid metabolism, and hepatocyte apoptosis (Chen P. et al., 2024). Therefore, this section discusses how NPs preserve hepatic mitochondrial homeostasis across diverse liver injury settings (Table 3).

**TABLE 3:** NPs regulating mitochondrial dysfunction in liver diseases.

Name	Source	Mw/Monosaccharide composition	Disease	Animals/Cells	Mitochondrial function	Mechanism/efficacy	Ref
POP	*Pleurotus ostreatus*	NG/Rha, Xyl, Man, Glc, Arab, Gal	CCl_4_-induced acute liver injury	Male Wistar rats/NG	Oxidative stress Mitochondrial apoptosis	POP → ↓ oxidative stress + ↓ mitochondrial apoptosis → alleviated CCl_4_-induced acute liver injury	(Zhu et al., 2019)
ASP	*Angelica sinensis*	NG/NG	5-FU-induced liver injury	Male C57BL/6J mice/human normal liver cell line MIHA	Mitochondrial apoptosis Oxidative stress	ASP → ↑ Nrf2/HO-1 antioxidant defense → ↓ ROS/oxidative DNA damage + ↓ Bax/Bcl-2 apoptosis → alleviated 5-FU-induced liver injury	(Zeng et al., 2021)
SMP	*Salvia miltiorrhiza* Bunge	NG/Man, Xyl, Arab, Glc, Gal	AFB1-induced liver injury	New Zealand meat rabbits/NG	Oxidative stress Mitochondrial apoptosis	SMP → ↑ Nrf2/HO-1/NQO1 signaling → ↓ oxidative inflammation + ↓ Cyt c–caspase-9/3 apoptosis → alleviated AFB1-induced liver injury	(Zhang L. et al., 2024)
DIP	*Dictyophora indusiata*	NG/D-Glc, D-Man, L-Rha, D-Gal, D-Xyl, L-Fuc	Arsenic-induced liver injury	SD rats/L-02 liver cells	Mitophagy Oxidative stress Mitochondrial apoptosis	DIP → ↓ ROS → restored ΔΨm + ↓ excessive PINK1/Parkin-mediated mitophagy → alleviated As-induced hepatotoxicity	(Hu et al., 2022) (Yan et al., 2024)
PS	*Coprinus comatus*	NG/NG	Alcoholic liver injury	Male SD rats/NG	Mitochondrial membrane integrity Mitochondrial apoptosis	PS → preserved mitochondrial membrane integrity and ultrastructure → ↓ lipid droplets and hepatocyte vacuolization → improved alcoholic liver injury	(Ozalp et al., 2014)
CS-PS	*Cordyceps sinensis* mycelia	NG/Arab, Glc, Gal	CCl_4_-induced liver fibrosis	Male Wistar rats/NG	Mitochondrial apoptosis	CS-PS → restored mitochondrial Bcl-2/Bax/Bak balance → ↓ Cyt c–caspase-9/3 apoptosis → reduced fibrosis progression	(Tian et al., 2019)
APP	“Qinguan” apple pomace	8 kDa/Arab, GalA, Gal, Rha, Xyl, Glc	NAFLD-like hepatic steatosis	HFD-induced obese mice/HepG2 cells	Oxidative stress Mitochondrial energy metabolism	APP → ↓ mitochondrial ROS → restored ΔΨm and respiratory function → improved HFD-induced hepatic metabolic disorder	(Chen et al., 2017)
GGL0.05S1	*Gomphrena globosa*	83.9 kDa/Glc, Arab	MASH	CDA-HFD-fed male C57BL/6 mice/L02 cells and mouse primary hepatocytes	Oxidative stress Mitophagy	GGL0.05S1 → ↑ TXN antioxidant capacity + ↑ FAO/mitophagy → ↓ ROS/lipotoxicity/NLRP3 activation → alleviated MASH.	(Chen M. et al., 2025)
SePFP	*Pyracantha fortuneana*	NG/NG	NAFLD-related hepatic steatosis	Male C57BL/6J mice/Mouse primary hepatocytes	Mitochondrial β-oxidation	SePFP → AMPK/ACC regulation → ↑ mitochondrial fatty acid transport, β-oxidation and ketogenesis → ↓ hepatic lipid accumulation	(Hu Y. et al., 2023)

POP: *pleurotus ostreatus* polysaccharides; ASP: *angelica sinensis* polysaccharide; SMP: *salvia miltiorrhiza* polysaccharide; DIP: *dictyophora indusiata* polysaccharide; PS: *coprinus comatus* polysaccharide; CS-PS: *cordyceps sinensis* mycelial polysaccharide; APP: apple pomace polysaccharide; GGL0.05S1: *Gomphrena globosa* L. arabinoglucan; SePFP: Selenium-enriched polysaccharides from *Pyracantha fortuneana*. Fuc, fucose; Gal, galactose; Glc, glucose; Xyl, xylose; Man, mannose; Rha, rhamnose; Arab, arabinose; GlcA, glucuronic acid; GalA, galacturonic acid. “NG”: not given; “↑”: increase or upregulation; “↓”: decrease or downregulation.

In drug- and toxin-induced liver injury, NPs mainly protect hepatocytes by limiting mitochondrial oxidative stress, apoptotic signaling, and maladaptive mitophagy. *Pleurotus ostreatus* polysaccharides (POP) alleviated carbon tetrachloride (CCl_4_)-induced acute liver injury in rats by strengthening antioxidant defenses, reducing lipid peroxidation and metabolic disruption, and suppressing Bax/Cyt c/caspase-mediated mitochondrial apoptosis (Zhu et al., 2019). *Angelica sinensis* polysaccharide (ASP) attenuated 5-fluorouracil (5-FU)-induced hepatotoxicity by restoring Keap1/Nrf2/HO-1-dependent antioxidant signaling, decreasing ROS accumulation and oxidative DNA damage, alleviating mitochondrial swelling, and suppressing Bcl-2/Bax-associated apoptosis (Zeng et al., 2021). In aflatoxin B1 (AFB1)-induced liver injury, *Salvia miltiorrhiza* polysaccharide (SMP) activated the Nrf2/HO-1/NQO1 pathway, reduced oxidative and inflammatory injury, and inhibited Cyt c/caspase-9/caspase-3-mediated mitochondrial apoptosis (Zhang L. et al., 2024). In arsenic-induced hepatotoxicity, DIP alleviated liver injury by inhibiting excessive PINK1/Parkin-mediated mitophagy, with proteomic evidence indicating broad recovery of mitochondrial, oxidative, apoptotic, and metabolic pathways (Hu et al., 2022; Yan et al., 2024).

NPs also protect against alcoholic liver injury and fibrosis, in which mitochondrial respiratory dysfunction and hepatocyte apoptosis drive disease progression. In an alcoholic diet-fed rat model, *C. comatus* polysaccharide (PS) attenuated alcohol-induced liver injury by reducing hepatocyte vacuolization and enhancing mitochondrial Cyt c oxidase activity, indicating improved mitochondrial function (Ozalp et al., 2014). In CCl_4_-induced liver fibrosis, a polysaccharide-containing CGA formula composed of *Cordyceps sinensis* mycelial polysaccharide (CS-PS), gypenosides, and amygdalin ameliorated hepatic fibrosis by suppressing hepatocyte apoptosis, as shown by reduced Fas/TNF-R1 signaling, Bax/Bcl-2 expression, Cyt c release, and caspase-9/3 activation (Tian et al., 2019).

In metabolic liver disease, especially metabolic dysfunction-associated steatohepatitis (MASH) and hepatic steatosis, mitochondrial lipid metabolism and quality control are central therapeutic targets. Apple pomace polysaccharide (APP) improved hepatic metabolic dysfunction in HFD-fed mice and palmitate-loaded HepG2 cells by reducing oxidative stress, suppressing mitochondrial ROS accumulation, and restoring mitochondrial respiration (Chen et al., 2017). In a choline-deficient, L-amino acid-defined (CDA)-HFD-fed MASH model, arabinoglucan GGL0.05S1 from *Gomphrena globosa* flowers alleviated steatohepatitis by promoting mitophagy and fatty acid β-oxidation, while reducing oxidative stress, lipotoxicity, and NLRP3-mediated inflammation (Chen M. et al., 2025). Selenium-enriched polysaccharides from *Pyracantha fortuneana* (SePFP) reduced hepatic lipid accumulation in aging mice by facilitating mitochondrial fatty acid transport and enhancing β-oxidation and ketogenesis (Hu Y. et al., 2023).

Overall, NPs may preserve hepatic mitochondrial homeostasis through regulation of redox balance, bioenergetics, lipid metabolism, and quality control, although these effects vary across disease contexts. The opposite regulation of mitophagy observed in toxic liver injury and MASH suggests that its functional consequence depends on the underlying mitochondrial state. Moreover, findings from multi-component formulations such as CGA cannot be attributed specifically to the polysaccharide component.

## 6 Prospects and challenges of NPs as mitochondrial modulators

Current literature supports two broad conclusions regarding NPs and mitochondrial regulation (Wu et al., 2025). First, structurally diverse NPs can modulate signaling networks that converge on mitochondrial function. The highest tier of evidence comes from studies combining well-defined polysaccharide preparations with direct mitochondrial functional readouts, including oxygen consumption rate, respiratory chain complex activity, ATP production, and mitochondrial calcium dynamics. By contrast, studies relying solely on indirect markers such as total ROS levels, antioxidant enzyme activity, and Bcl-2/Bax or caspase expression support cytoprotective or cytotoxic outcomes but do not independently establish a mitochondrial mechanism. Second, the regulatory effects of NPs are inherently context-dependent. In neurodegenerative, metabolic, cardiovascular, and hepatic injury, NPs generally preserve mitochondrial bioenergetics and quality control to exert protective effects. In cancer, by contrast, selected NPs instead amplify mitochondrial stress or disable adaptive mitophagy to promote tumor cell death. This bidirectional, context-specific pattern distinguishes NPs from single-target mitochondrial agents and underpins their broad therapeutic potential.

Despite growing evidence, three core issues limit rigorous interpretation of the field and slow translational progress. First, direct versus indirect action remains poorly distinguished. Many high-molecular-weight oral polysaccharides exert systemic mitochondrial benefits indirectly, via gut microbiota remodeling, immune receptor activation, microbial metabolite production, or improved metabolic homeostasis, rather than by directly entering cells and targeting mitochondria. These indirect mechanisms are pharmacologically valuable but require distinct biomarkers, dosing strategies, and development pathways. The term “mitochondria-targeting” should be reserved for agents with demonstrated organelle exposure or direct functional interaction with mitochondrial systems. Second, structure–activity attribution remains preliminary. As discussed in Section 2, structural heterogeneity among polysaccharide preparations complicates the identification of clear structure–activity relationships. Definitive analysis requires fraction libraries, orthogonal structural characterization, blinded batch comparisons, and exposure-normalized pharmacological assays. Third, evidence quality is uneven across studies. Most investigations rely on indirect pathway markers rather than direct mitochondrial functional measurements, and many studies use supraphysiological doses that may reflect nonspecific toxicity rather than targeted regulation. Claims regarding mitophagy often lack flux assessment, and ROS-related studies rarely specify oxidant species or quantify specific damage products.

To advance NPs from descriptive bioactivity screening toward mechanism-guided translational therapeutics, several research priorities should be addressed. First, standardized structural characterization and quality control are foundational. Each study should fully report biological source, batch information, Mw distribution, key glycosidic linkages, monosaccharide composition, and contaminant levels to ensure reproducibility and enable cross-study comparison. Second, mechanism-oriented mitochondrial functional validation should become standard. Studies claiming mitochondrial effects should include at least one direct functional assay, such as respiratory phenotyping, membrane potential measurement, or calcium dynamics analysis. Mitophagy claims should assess flux rather than static protein levels, and mechanistic conclusions should be supported by inhibition, knockdown, or rescue experiments. Third, emerging technologies may provide deeper mechanistic insight. Single-cell transcriptomics and spatial metabolomics may resolve cell- and tissue-specific mitochondrial responses to polysaccharides, whereas mitochondrial proteomics and integrated multi-omics may improve identification of target-engagement pathways. Organoid models may provide more disease-relevant experimental systems, while AI-assisted approaches may facilitate structure–activity analysis, candidate screening, and precision medicine strategies. Fourth, delivery optimization and pharmacokinetic evaluation are needed to bridge *in vitro* and *in vivo* findings. Mitochondria-targeted peptides, hybrid nanoparticles, and structural modifications may enhance cellular uptake, tissue targeting, and blood–brain barrier penetration (Li et al., 2024b). Pharmacokinetic studies are needed to confirm whether these strategies improve tissue exposure and mitochondrial delivery *in vivo* without compromising safety. Fifth, clinical translation requires stronger human evidence. polysaccharide-K and lentinan have been used regionally as adjunctive cancer therapies, while fucoidan has undergone preliminary clinical evaluation (Narayanan et al., 2023; Yoshino et al., 2016; Tsai et al., 2017). However, clinical findings remain inconsistent and mainly concern survival, disease control, and safety rather than mitochondrial function. As summarized in Table 4, mitochondrial effects are supported predominantly by preclinical studies, and none of these polysaccharides has been clinically validated as a mitochondrial modulator. Future trials should use standardized preparations and include direct mitochondrial endpoints. Clinical translation will also require clear regulatory frameworks, GMP-compliant manufacturing and quality-control systems, and scalable production strategies for broader clinical use.

**TABLE 4:** Clinical evidence and mitochondrial translational gaps of representative NPs.

Polysaccharide	Clinical evidence	Preclinical mitochondrial evidence	Main limitation	Ref
Polysaccharide-K	Regionally used as an oncology adjuvant in Japan. Gastric cancer trials and meta-analysis suggested a modest survival benefit	Mitochondrial function was not directly assessed in the clinical studies	Evidence is based mainly on older regional treatment regimens, and the proposed clinical mechanism is predominantly immunomodulatory	(Narayanan et al., 2023)
Lentinan	Regionally used as an oncology adjuvant in Japan and China. Earlier pooled evidence suggested a survival benefit, whereas a later Phase III trial showed no improvement in overall survival	Preserved mitochondrial integrity and reduced apoptosis through CAV1/SDHA regulation in diabetic mice	Clinical and mitochondrial evidence derives from different diseases, and no clinical mitochondrial endpoints have been evaluated	( Yoshino et al., 2016; Hu S. et al., 2023 )
Fucoidan	Not approved as an anticancer drug. A small colorectal cancer trial improved disease control rate but not overall or progression-free survival	Improved respiratory-chain activity, ATP production, mitochondrial redox balance, biogenesis, mtDNA stability, and mitophagy in cellular and animal models	Clinical evidence is preliminary; preparations are heterogeneous, and mitochondrial target engagement has not been demonstrated in humans	( Tsai et al., 2017; Ji et al., 2022; Jing et al., 2021; Zhao et al., 2021 )

In summary, mitochondrial dysfunction is a common feature of many major diseases, making the maintenance of mitochondrial homeostasis an important therapeutic strategy. NPs can modulate multiple aspects of mitochondrial function, including bioenergetics, redox balance, mitochondrial quality control, calcium homeostasis, and apoptosis, with effects that vary across disease contexts. However, robust structure–activity relationships, direct mitochondrial actions, and clinical efficacy remain insufficiently established. Further progress will require standardized polysaccharide preparations, direct mitochondrial functional validation, and clinically relevant studies.

## Glossary

ATP: Adenosine triphosphate
AGP: *Aloe* gel glucomannan
AD: Alzheimer’s disease
ACPS: *Amanita caesarea* polysaccharides
AMPK: AMP-activated protein kinase
ABPA1: An acetylated mannan isolated from *Aloe vera*
ASP: *Angelica sinensis* polysaccharide
ARPs: *Anoectochilus roxburghii* polysaccharides
ACP: *Antrodia camphorata* polysaccharide
APP: Apple pomace polysaccharide
ASKP: *Artemisia sphaerocephala* Krasch polysaccharide
APS: *Astragalus* polysaccharides
BAT: Brown adipose tissue
CHS2: A sulfated polysaccharide from *Chaetomorpha linum*
CPS: Capsular polysaccharide
CVD: Cardiovascular diseases
CPP: *Codonopsis pilosula* polysaccharide
PS: *Coprinus comatus* polysaccharide
CCP: *Coptis chinensis* polysaccharide
CS-PS: *Cordyceps sinensis* mycelial polysaccharide
CYP: *Corydalis yanhusuo* polysaccharide
PCCL: *Cuscuta chinensis* Lam polysaccharide
Cyt c: Cytochrome c
DSP: Danshen polysaccharides
DOP: *Dendrobium officinale* polysaccharides
DIP: *Dictyophora indusiata* polysaccharide
DOX: Doxorubicin
Drp1: Dynamin-related protein 1
ETC: Electron transport chain
EPP: *Enteromorpha prolifera* polysaccharide
Fis1: Mitochondrial fission 1 protein
GEP: *Gastrodia elata* polysaccharide
GGL0.05S1: *Gomphrena globosa* L. arabinoglucan
GLP: *Ganoderma lucidum* polysaccharides
GP4: 4.7-kDa ginseng polysaccharide
GSH-Px: Glutathione peroxidase
HFD: High-fat diet
HO-1: Heme oxygenase-1
IPS1 and IPS2: Polysaccharides from *Paecilomyces cicadae* TJJ1213
I/R: Ischemia/reperfusion
iWAT: Inguinal white adipose tissue
Keap1: Kelch-like ECH-associated protein 1
LBP: *Lycium barbarum* polysaccharides
MASH: Metabolic dysfunction-associated steatohepatitis
MCP: *Momordica charantia* polysaccharide
Mfn1/Mfn2: Mitofusin 1/2
MLP: Mulberry leaf polysaccharides
MPs: Maca polysaccharides
mPTP: Mitochondrial permeability transition pore
**mtCa** ^**2+**^: Mitochondrial calcium
mtDNA: Mitochondrial DNA
mTOR: Mammalian target of rapamycin
Mw: Molecular weight
NAFLD: Non-alcoholic fatty liver disease
NG: Not given
NPs: Natural polysaccharides
Nrf2: Nuclear factor erythroid 2-related factor 2
OHSS2: *Cladophora oligoclada* sulfated polysaccharide
OPA1: Optic atrophy 1
OXPHOS: Oxidative phosphorylation
PCP: Pocoa polysaccharides
PD: Parkinson’s disease
PGC-1α: Peroxisome proliferator-activated receptor γ coactivator 1α
PI3K: Phosphoinositide 3-kinase
PINK1: PTEN-induced putative kinase 1
POP: *Pleurotus ostreatus* polysaccharides
PPe-s: Pumpkin polysaccharide hydrolysates
PSG: *Ganoderma atrum* polysaccharide
PSP: *Polygonatum sibiricum* polysaccharides
RGP: Red ginseng polysaccharide
ROS: Reactive oxygen species
SDFP-2: Dragon fruit polysaccharide
Se-GTP: Selenium-enriched green tea polysaccharides
SePFP: Selenium-enriched polysaccharides from *Pyracantha fortuneana*
SFG: Sulfated fucogalactan
SIRT1: Silent information regulator 1
SMP: *Salvia miltiorrhiza* polysaccharide
SOD: Superoxide dismutase
TFP: *Tremella fuciformis* polysaccharide
TPSP2: Purified polysaccharide TPSP2 from Tremella fuciformis
UCP1: Uncoupling protein 1
WTRP-A0.2b: Pectic polysaccharide from *Typhonii* Rhizoma
XQZ3: Acidic polysaccharide from *Chlorella pyrenoidosa*
ΔΨm: Mitochondrial membrane potential
6-OHDA: 6-hydroxydopamine
